# Association of Interleukin-1*α* Functional Polymorphism with Risk of Chronic Periodontitis in Han Chinese Population

**DOI:** 10.1155/2021/6614835

**Published:** 2021-03-27

**Authors:** Xiaowei She, Hua Xiao, Shuang Lu, Lijun Guo

**Affiliations:** ^1^Department of Stomatology, The First Affiliated Hospital of Yangtze University, Jingzhou, Hubei 434000, China; ^2^Hubei College of Chinese Medicine, Jingzhou, Hubei 434020, China

## Abstract

Chronic periodontitis (CP) is a common inflammatory illness affecting a large proportion of humans. Genetic factors are thought to play important roles in its onset and development. A functional polymorphism (rs1800587) in the promoter of the interleukin-1*α* gene (−889 C/T) has been found to confer risk of CP primarily in Europeans, but the association between this variant and CP in the Chinese population remains less conclusive. In the current study, we aimed to investigate the association between rs1800587 and CP in case-control samples of Han Chinese origin. A total of 1,777 study subjects, including 884 CP patients and 893 healthy controls, were collected. Genotyping of rs1800587 was performed using the SNAPSHOT method, and statistical analyses were conducted to evaluate the association between rs1800587 and CP. In our sample, rs1800587 was significantly associated with the onset of CP (additive model, T-allele vs. C-allele, *p* = 0.00359, odds ratio = 1.446, 95% confidence intervals (CIs) = 1.127–1.855; dominant model, (TT + TC) vs. CC, *p* = 0.00250, odds ratio = 1.502, 95% CIs = 1.152–1.957; overdominant model, TC vs. (TT + CC), *p* = 0.00264, odds ratio = 1.508, 95% CIs = 1.152–1.976). The T-allele and [TC] genotypes of rs1800587 were significantly overrepresented in CP patients compared with controls. Our data suggest that rs1800587 of IL-1*α* is significantly associated with the risk of CP in Han Chinese subjects, further confirming its possible involvement in the disease.

## 1. Introduction

Periodontitis is a common inflammatory illness and a major cause of tooth loss in adults and seniors [1]. Periodontitis is traditionally classified as either aggressive periodontitis (AgP) or chronic periodontitis (CP) [2]. AgP is a rare disease affecting about 0.1% of general populations [3], whereas CP is a common form of the disease characterized by chronic inflammatory responses to oral bacteria (both commensal and pathogenic). Overall, periodontal health problems bring significant social and economic burden. For example, the severe generalized form of CP is a major threat to oral health (especially in senior adults) and life quality [4, 5] and affects about 8% to 13% of individuals [6]. The unneglectable link between CP and a series of diseases and complications (e.g., Alzheimer's disease [7], head and neck squamous cell carcinoma [8], coronary heart disease [9], and negative pregnancy outcomes [10]) also calls for effort in scientific investigation and clinical management. Evidence suggests that both oral pathogens and inflammatory burden are involved in CP and its relevant complications [11–13]. Genetic and environmental modifiers, which largely modulate host-response to pathogens and inflammation, are thus likely important factors influencing the disease.

Up to now, multiple environmental factors (e.g., smoking and obesity) and age are found to affecting the risk of CP [14]. Notably, earlier twin studies [15] and epidemiological reports of familial aggregation of severe CP [16, 17] suggest a genetic component in its pathogenesis, although the proportion of the disease risk attributable to genetic factors is still under debate. Candidate gene studies have been conducted to clarify the genetic and biological basis of CP, with an emphasis on genes encoding molecules relevant to host immune responses (e.g., pattern recognition receptors and associated molecules, inflammatory mediators and their receptors, and enzymes) [18]; for example, genes encoding interleukin-1 (IL-1) family proteins are thought to play pivotal roles in CP [19]. The IL-1 family proteins mainly refer to the well-characterized cytokines IL-1*α*, IL-1*β*, the IL-1 receptor antagonist IL-1ra, and their two membrane receptors IL-1RI and IL-1RII. They are well-known key regulators in both innate and adaptive immunity and have been highlighted in a variety of infectious and autoimmune diseases, as well as in systemic inflammatory responses [20]. IL-1*α* is generally recognized as a proinflammatory mediator facilitating the pathogenesis of multiple infectious diseases [21], and genetic variations in L-1*α* are found to be associated with the onset and progression of CP. Specifically, the SNP rs1800587 (also known as IL-1*α* (−889 C/T)) is significantly associated with CP but not AgP [22, 23]. For instance, meta-analyses of association studies for CP and an IL-1*α* SNP, rs1800587, reveal that this SNP is significantly associated with the risk of CP [24–26]. Therefore, rs1800587 likely plays an important role in CP pathogenesis.

Nevertheless, most of the studies of rs1800587 in CP were performed in Europeans. Given that the genetic architecture of disease is strongly affected by different genetic backgrounds and population histories between ethnic groups, whether rs1800587 is also associated with CP in other populations is an urgent question to investigate. So far, there have been several studies investigating the association between this SNP and CP in Chinese subjects, but the sample sizes were relatively small, resulting in inconsistent conclusions [27–31]. Considering that the minor T-allele frequency of rs1800587 in Han Chinese is relatively low (∼7%), larger samples are necessary to obtain reliable results. We therefore recruited 884 CP cases and 893 controls of Han Chinese ancestry for genotyping of rs1800587. By analyzing the association between rs1800587 and CP in a relatively large Han Chinese sample, we sought to gain insights into the potential involvement of this variation in CP pathogenesis.

## 2. Materials and Methods

### 2.1. Study Subjects

A total of 884 CP patients and 893 healthy controls aged from 35 to 65 years were recruited under a study protocol subjected to the Declaration of Helsinki ethical guidelines (version 2013), and the protocol was approved by the Ethical Committee of the First Affiliated Hospital of Yangtze University (Approval number 20201202). All participants have signed the informed consent. They were all unrelated Han Chinese with residency in the Hubei Province and enrolled in the current study during their dental visits at the First Affiliated Hospital of Yangtze University between January 2014 and January 2019. The cases and controls were primarily age- and gender-matched. All participants were evaluated as being in good general health, and smoking statuses were recorded by interviewing. They all have at least 20 teeth in the mouth, and CP was diagnosed based on the AAP 1999 classification [2] according to the criteria combining clinical indices, pocket depth (PPD) and bleeding on probing, clinical attachment loss (CAL), tooth mobility, gingival recession, supplemented with physical examination, and dental and medical history review. Specifically, PPD and CAL were measured at 6 points around each tooth. Subjects were considered orally healthy when they have a probing depth of ≤3 mm, no signs of gingival inflammation, and meanwhile no history of periodontal diseases [2]. To avoid confounders in the etiological attributes of CP, subjects were excluded from further enrollment if they (1) were pregnant or lactating; (2) had used any antibiotics or anti-inflammatory medications during the past six months; (3) were affected by any severe medical complications influencing periodontal health such as rheumatoid arthritis, diabetes, and osteoporosis; or (4) received periodontal treatment within the past six months. Baselines of the clinical indices are listed in Table 1.

### 2.2. DNA Separation

Saliva was collected from the participants, and genomic DNA was then extracted according to the manufacturer's protocol. DNA was extracted from these saliva samples and then dissolved in 50 *μ*L TE buffer (10 mM Tris (pH 7.8) and 1 mM EDTA), and the concentrations were estimated by measuring the optical densities at 260.

### 2.3. SNP Genotyping

The SNAPSHOT method was applied for genotyping of rs1800587 following the manufacturer's protocol. The PCR primers were designed following a previous study [32], and the primer sequences were 5'-AAGCTTGTTCTACCACCTGAACTAGGC-3' (forward) and 5'-TTACATATGAGCCTTCCATG-3' (reverse). DNA fragments containing rs1800587 were amplified from the total genomic DNA from each subject in 10 *μ*L polymerase chain reaction (PCR) mixture. The 10 *μ*L reaction mixture was comprised of 1 *μ*L template DNA (10 ng) and 9 *μ*L reaction mixture containing 7 *μ*L PNM, 1 *μ*L 10 × PCR buffer, 0.5 *μ*L 25 mmol/L MgCl_2_, and 0.2 U Taq polymerase (Takara). The PCR was then performed with an ABI 9700 cycler using the program as follows: 5 min denaturation at 95°C, followed by 40 cycles of the sequential reactions at 95°C for 30 s, 60°C for 30 s and then 30 s at 72°C, and eventually a final extension step at 72°C for 10 min. The PCR products were purified with SAP and treated with Exo-I exonuclease. SNAPSHOT primers were specifically designed for amplifying the site of the target SNP. The reaction lasted for one base extension, and the products were then loaded onto an ABI 3730 automatic sequencer for genotype callings of rs1800587. The genotype callings were then verified manually. All the experiments and data analyses were performed in a double-blinded manner and were independently reviewed by two authors. The analyses were also reconducted in 5% of the sample (randomly selected) to ensure a 100% concordance of the results.

### 2.4. Statistical Analyses

Power analysis was performed using the Power and Sample Size Program software [33], and the commonly observed OR of 1.50 was applied in the power analysis, which corresponds to a “medium” gene effect. Hardy–Weinberg equilibrium was tested for case and control samples through *χ*^2^ tests on Stata 14 (Stata Corp., TX, USA). Descriptive statistical analysis treating the genotyping results as categorical variables was performed. Allelic and genotypic tests were carried out to obtain single marker-based association results through *χ*^2^ tests.

## 3. Results

The demographic information including age, gender, smoking status, and others of our sample is presented in Table 1. No significant differences between CP cases and controls were observed for age (*p* = 0.649), gender ratio (*p* = 0.107), or smoking status (*p* = 0.546). Ideal genotyping call rate was obtained for rs1800587, and the genotype frequencies of rs1800587 were confirmed to be in Hardy–Weinberg equilibrium in both cases and controls (*p* = 0.837 in CP cases and *p* = 0.405 in controls).  Table 2 shows the allelic and genotypic distributions of rs1800587 in both CP cases and controls. Briefly, T-allele of rs1800587 was carried by 7.74% (275/3554) of the overall participants, and 0.62% (11/1777) of them were homozygous. Before performing the statistical analysis, we conducted a power analysis on our total sample size using the following assumptions: 884 CP cases and 893 controls, two-tailed *α* = 0.05, and a commonly observed OR (1.50) in genetic association studies (corresponding to a “medium” gene effect). Under the additive genetic model, given the frequency of T-allele in control Chinese populations (0.064), the present sample size revealed a 62.4% power of detecting a significant association; under the dominant genetic model, given the frequency of [T/T + C/T] genotype in control Chinese populations (0.123), the present sample size revealed a 85.4% power of detecting a significant association.

A significant increase of the T-allele frequency of rs1800587 in our Han Chinese CP patients compared with healthy controls was observed (T-allele, 0.0905 in cases and 0.0644 in controls, Table 2). Therefore, the minor T-allele of rs1800587 was significantly associated with the risk of CP in Han Chinese (allelic model, *p* = 0.00359, odds ratio = 1.446, and 95% confidence intervals (CIs) = 1.127–1.855; Table 2). Significant difference was also observed in the distributions of different genotypes of this SNP between CP patients and controls (additive model: *p* = 0.00382, odds ratio = 1.446, and 95% CIs = 1.126–1.856; Table 2). Again, the frequency of the [T/T + C/T] genotypes was 17.42% in CP patients and 12.32% in controls, suggesting a significant overrepresentation of the T-allele in cases (dominant model: *p* = 0.00250, odds ratio = 1.502, and 95% CIs = 1.152–1.957; Table 2). However, we did not observe a significant difference of [TT] genotype frequency between CP cases and controls (recessive model: *p* = 0.75, odds ratio = 1.214, and 95% CIs = 0.369–3.991; Table 2), while the frequency of [TC] genotype was significantly higher in CP cases than in controls (overdominant model: *p* = 0.00264, odds ratio = 1.508, and 95% CIs = 1.152–1.976; Table 2).

## 4. Discussion

As a chronic inflammatory disorder, the pathology of CP is considered closely related to the production and activation of a series of inflammatory mediators including interleukins, which are primarily synthesized and produced by immune cells such as lymphocytes, monocytes, macrophages, and epithelial cells. [20]. Specifically, the elevated presence of IL-1 family proteins, including both the cytokines and the receptors, has been found to participate in the oral host-responses to microbial infection, as well as in extracellular matrix catabolism, bone resorption, and periodontal tissue remodeling [19, 34–36]. In the hypothesized gene-environment interaction model for the etiology of CP, it is believed that genetic variations affecting the production and/or function of the interleukins may cause altered immune responses following periodontal infection and thereby lead to different levels of risk of developing this disease [21, 37].

Among genetic variations explored so far in CP, rs1800587 has caught wide attention. So far, rs1800587 has been investigated for its associations with CP in multiple world populations, such as Africans, Europeans, and Americans [22, 24–26, 38–41]. Considering that genetic analysis results can often be inconsistent across populations due to ethnic-specific genetic background features, it is of great interest to examine the association between rs1800587 and CP in Chinese samples with sufficient statistical power. It is intriguing that rs1800587 shows significant association with CP in many samples from different populations. Despite the divergence in the allele frequencies of rs1800587 in distinct populations (e.g., the T-allele frequency ranges from 28.7% to 40.4% in Europeans and Africans, while the T-allele frequency is 7.74% in our Han Chinese sample), both the previous meta-analyses of CP in Europeans and Africans [24, 25] and our study has found a disproportional overrepresentation of the T-allele of rs1800587 in CP patients compared with controls. However, the effect sizes of rs1800587 to CP are different across different ethnic groups, likely due to its varied allele frequencies. Nevertheless, it is noteworthy that analyses of the association between rs1800587 and CP in previous small Han Chinese cohorts [27–31] obtained mixed results, probably owing to their small sample sizes. Another potential explanation is that these previous samples were recruited at different regions in China, and genetic architecture differences among Chinese subjects originated from different areas are widely evident [42, 43]. It is also possible that certain environmental or socioeconomic factors that differ between different regions may have affected the role of this SNP in the pathogenesis of CP. However, this hypothesis cannot be tested at present as such information (environmental exposures, socioeconomic factors, etc.) is not available for all samples.

Alongside the genetic evidence, the potential functional impact of rs1800587 (especially the CP risk T-allele) on inflammatory responses involved in CP has also be confirmed. Rs1800587 locates at the position −889 upstream of the IL-1*α* translation start site. Its surrounding genomic region is hypothesized to facilitate DNA accessibility based on the previously published DNase hypersensitivity data by ENCODE. Besides, rs1800587 colocalizes with the RFX1 protein motif, suggesting that this SNP may lead to altered cell physiology relevant to RFX1 [44]. Recent data suggest that different alleles of rs1800587 lead to differential DNA binding of multiple transcription factors, such as the AP-1 family proteins [45]. Intriguingly, the allelic imbalance is significantly enhanced when the cells are immune-activated with lipopolysaccharide (LPS) treatment. Specifically, the [T/T] genotype of rs1800587 significantly increases the promoter activity of the IL-1*α* gene compared with the [C/C] genotype in mononuclear cells following LPS stimulation [46], and Wei et al. have observed significantly elevated transient promoter-luciferase activity in human astroglial cells carrying [T/T] genotype at rs1800587 [47]. Similarly, cells carrying the [T/T] genotype at rs1800587 exhibit an almost fourfold increase in the IL-1*α* protein levels [36]. Therefore, rs1800587 might affect the inflammatory processes related to IL-1*α*. More intriguingly, Loo et al. previously found that the protein expressions of IL-1*α* (measured by ELISA) were significantly higher in CP cases than in controls of the Chinese population [30], adding further evidence supporting that the T-allele of rs1800587 might contribute to the risk of developing CP by modulating IL-1*α* levels. Additionally, polymorphisms in the IL-1 gene cluster on chromosome 2 are also correlated with the risk of severe periodontitis in adults [34]. Therefore, whether rs1800587 contributes to the pathogenesis of CP via modulating the production of IL-1*α* is a question of specific interest for future studies.

### 4.1. Limitation

Despite the identification of genetic predisposing factors of CP, the current study has several limitations. As the sample recruitment was initiated before the publication of the new guidelines for periodontitis in 2017, the current study mainly focuses on the traditionally defined “CP”. Further analyses in larger Han Chinese samples of different types, stages, and grades of the disease are therefore necessary. In addition, radiograph should also be conducted for all participants if possible, which is unfortunately missing for a great portion of cases in the current study, as radiographic bone loss and tooth loss could provide valuable evidence for the disease progression and severity. Similarly, socioeconomic indices should be collected for the participants, as oral hygiene can be jeopardized by insufficient income, limited access to healthcare, and so on. Additionally, although the sample size is relatively larger compared with some earlier studies (especially in Chinese), further validation of the associations between rs1800587 and CP in an independent cohort is needed.

## 5. Conclusion

Our study suggests that rs1800587 in the IL-1*α* gene is a risk variant for CP in the Chinese population. This result has promoted our understanding of the genetic mechanisms of this illness in Han Chinese, and further studies focusing on detailed mechanisms underlying this polymorphism and the gene in CP and other periodontal conditions are needed. For example, do the differences in production and function of IL-1*α* lead to altered inflammatory responses following microbial infection? Hopefully addressing these problems will provide valuable insights for the understanding, treatment, and prevention of CP.

## Figures and Tables

**Table 1:** Demographic and clinical characteristics of the cases and controls.

Variables	Case (*n* = 884)	Control (*n* = 893)	*p* value
Age (years) (mean ± SD)	50.24 ± 9.05	50.04 ± 8.74	0.649
*Gender (%)*			
Male	513	495	0.107
Female	371	398	
*Smoker*			
Yes	468	465	0.546
No	416	428	
No. of lost teeth (mean ± SD)	1.93 ± 1.40	0.47 ± 0.50	<0.0001
BOP (%)	81.17 ± 12.83	12.23 ± 7.11	<0.0001
PPD (mm) (mean ± SD)	6.39 ± 0.79	1.18 ± 0.45	<0.0001
CAL (mm) (mean ± SD)	5.89 ± 0.93	1.24 ± 0.98	<0.0001

SD: standard deviation; BOP: bleeding on probing; PPD, probing pocket depth; CAL: clinical attachment loss.

**Table 2:** Genotype and allele distributions of rs1800587 in the cases and controls and their associations with risk of CP.

		Case (*n* = 884)	Control (*n* = 893)	*p* value	Effect group	OR	95% CIs
Allelic	T-allele	160	115	0.00359	T-allele	1.446	1.127–1.855
	C-allele	1608	1671				

Additive	TT	6	5	0.00382	T-allele	1.446	1.126–1.856
	TC	148	105				
	CC	730	783				

Dominant	TT + TC	154	110	0.0025	TT + TC	1.502	1.152–1.957
	CC	730	783				

Recessive	TT	6	5	0.75	TT	1.214	0.369–3.991
	TC + CC	878	888				

Overdominant	TC	148	105	0.00264	TC	1.508	1.152–1.976
	TT + CC	736	788				

Codominant	TT	6	5				
	TC	148	105	1.00	TT	0.851	0.253–2.863
	CC	730	783	0.68	TT	1.287	0.391–4.236

OR, odds ratio; CIs, confidence intervals.

## Data Availability

All the data generated in this study are available from the corresponding author (Hua Xiao) upon request.

## Abbreviations

CP: Chronic periodontitis
AgP: Aggressive periodontitis
IL-1: Interleukin-1
SNPs: Single-nucleotide polymorphisms
LPS: Lipopolysaccharide
CIs: Confidence intervals
PPD: Pocket depth
CAL: Clinical attachment loss
PCR: Polymerase chain reaction
SD: Standard deviation
BOP: Bleeding on probing
OR: Odds ratio.
